# Therapeutic potential of flavonols in the treatment of JCPyV

**DOI:** 10.1128/spectrum.01527-25

**Published:** 2025-08-21

**Authors:** Evan MacLure, Kaitlin Garabian, Bethany A. O'Hara, Wenqing Yuan, Jacob Kaiserman, Avraham S. Lukacher, Sheila A. Haley, Walter J. Atwood

**Affiliations:** 1 Department of Cell Biology, Biochemistry, and Molecular Biology, Brown University 736725 , Providence, Rhode Island, USA; University of Manitoba, Winnipeg, Canada

**Keywords:** polyomavirus, JCPyV, PML, CNS, flavonol, antiviral

## Abstract

JC polyomavirus (JCPyV) is a double-stranded DNA virus that causes progressive multifocal leukoencephalopathy (PML), a demyelinating disease with a high rate of morbidity and mortality. There are limited treatment options for patients diagnosed with PML, and most involve reconstitution of immunological functions. In this paper, we show that flavonols, specifically quercetin, myricetin, and fisetin, significantly reduce JCPyV infection in human glial cells. We further show that quercetin and fisetin can suppress viral spread in an already established infection, suggesting therapeutic viability of these drugs as antivirals. Additional research is required to determine the mechanism of action of flavonols in inhibiting JCPyV infection and to investigate whether these compounds have the potential to cross the blood-brain barrier in significant amounts to inhibit the virus in the brain parenchyma.

**IMPORTANCE:**

The human polyomavirus, JC polyomavirus (JCPyV), causes significant morbidity and mortality in immunosuppressed or immunomodulated patients. There are no approved antivirals to treat JCPyV-induced disease. Flavonols are naturally occurring polyphenolic compounds that are known to antagonize pathways involved in JCPyV infection. Several flavonols were found to inhibit initial JCPyV infection and viral spread in a glial cell line and in normal human glial cells. These represent a promising new treatment paradigm for patients at risk of suffering from JCPyV-induced disease.

## INTRODUCTION

JC polyomavirus (JCPyV) is a non-enveloped double-stranded DNA virus and causative agent of progressive multifocal leukoencephalopathy (PML), a fatal demyelinating disease (1–3). Approximately 60–80% of the world population is infected with JCPyV according to seroepidemiological studies, with the likelihood of acquiring the virus increasing with age (4). JCPyV is found in high concentrations in raw sewage and is believed to spread through the fecal-oral route (5, 6). Following infection, the virus establishes a lifelong persistent infection in the kidneys and is asymptomatic (7). In a small subset of cases, usually involving prolonged immunosuppression or immunomodulation, JCPyV traffics to the central nervous system and causes PML (8, 9). JCPyV likely penetrates the brain parenchyma by invading across the blood-CSF and blood-brain barriers (BBB) (10, 11). Once in the CNS, the virus infects astrocytes and oligodendrocytes, leading to the formation of multifocal white matter lesions characteristic of PML (4, 12, 13). PML has a high degree of morbidity and mortality, and most survivors are left with permanent focal neurological deficits (1, 3, 14, 15).

The neuropathology that defined PML was initially described in 1958 in brain tissue from patients diagnosed with chronic lymphocytic leukemia and Hodgkin’s lymphoma experiencing neurological symptoms (16, 17). PML remained extremely rare until the AIDS pandemic, when PML developed in approximately 5% of patients due to immunosuppression caused by the HIV-1-induced loss of CD4^+^ T cells (18). Restoration of immune function by antiretroviral therapy (ART) in these patients prevented further neurological dysfunction, and this approach remains the only effective therapeutic in the treatment of PML in AIDS patients (19). However, immediate reconstitution of the immune system by ART can lead to immune reconstitution inflammatory syndrome (IRIS) (19, 20). IRIS involves local inflammation around CNS parenchyma from infiltration of JCPyV-specific T cells and is often fatal if not managed appropriately (21, 22).

Due to the high rates of morbidity and mortality in patients with PML, the search for a potent therapeutic is of utmost importance. Current areas of research involve the use of ART therapies, nucleoside analogs (cytarabine, cidofovir, brincidofvir, and leflunomide), HT-2A serotonin receptor blockers (chlorpromazine, risperidone, and mirtazapine), retrograde trafficking inhibitors, and MAPK-ERK inhibitors (23, 24). Cytarabine has been shown to reduce JCPyV infection *in vitro* but led to increased viral loads in patients (25, 26). Cidofovir was similarly found to lack a significant reduction in JCPyV viral load and was not more beneficial in combination with ART administered therapies (27, 28). Brincidofovir is a lipid-ester derivative of cidofovir and was found to reduce JCPyV DNA copy number at 100× smaller concentrations than cidofovir (23, 29). Leflunomide was found in a small clinical study to improve symptoms in one PML patient with tapering of an immunomodulatory medication, though results are inconclusive (30–32). 5-HT2R inhibitors have shown mixed results in improving disease outcomes in PML. Mirtazapine has been shown to improve PML symptoms in combination with mefloquine and boasts improved cognitive functioning 3–6 months post-treatment, but other data show no significance (33–35). Chlorpromazine failed to reach therapeutically relevant half-maximal effective concentration (EC50) *in vivo* to establish a therapeutic effect demonstrated *in vitro* (36). Retro-2(cycl) is a retrograde trafficking inhibitor that has broad inhibition of polyomavirus transport to the endoplasmic reticulum but has not been investigated *in vivo* (37). Recently, a MAPK-ERK inhibitor, GW-5074, has been found to significantly reduce JCPyV infection and spread *in vitro*, suggesting connections between inhibiting cell proliferation and inhibiting viral infection (38).

**Fig 1:**
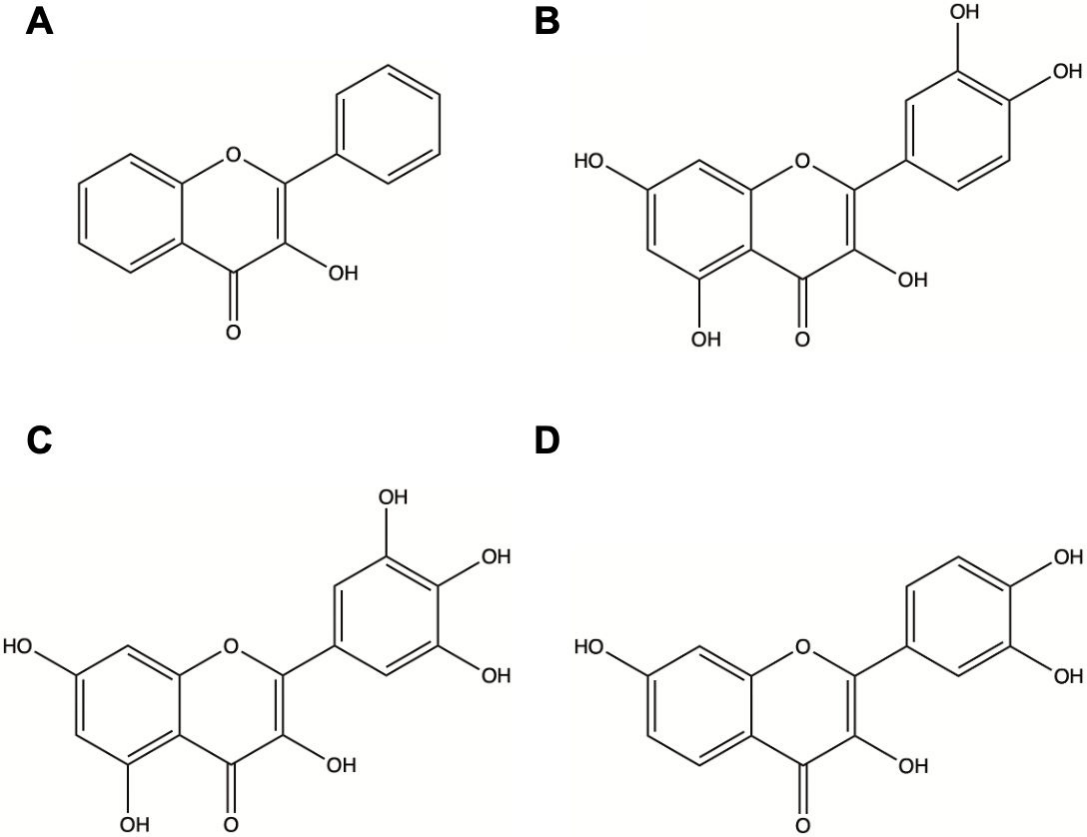
(**A**) General structure of a flavonol. (**B**) Structure of quercetin. (**C**) Structure of myricetin. (**D**) Structure of fisetin.

Flavonols are a subclass of flavonoids derived naturally from plants and bacteria and are frequently found in fruits, vegetables, and tea (39). Flavonols are distinct from other flavonoids due to a double bond between C2 and C3 and a carbonyl on C4, which creates a highly conjugated pi system (40). Flavonols are also highly glycosylated, which can affect solubility and biological effects (41). Quercetin, myricetin, and fisetin, which are the subjects of this study, are chemical analogs that differ in the hydroxyl arrangement around the polyphenolic core and are subject to different patterns of glycosylation (Fig. 1A through D). Due to the polyphenolic ring structure, flavonols encompass a wide range of functions including anti-inflammatory, antiviral, anticarcinogenic, and antioxidant properties (39, 42, 43). Flavonols have been associated with regulation of the PI3K-AKT-mTOR survival pathway and the MAPK/ERK pathway by inhibiting the phosphorylation of these key proteins (44–47). Each flavonol of interest was also found to inhibit the G_1_/S phase transition in the cell cycle and thus maintain the cells in interphase (48–50). Flavonols have also been shown to be pro-survival as they induce apoptosis and autophagy (51). Quercetin, myricetin, and fisetin also induce anti-inflammatory functions through inhibitory modulation of the NF-kB pathway that is responsible for the secretion of various pro-inflammatory cytokines, including TNF-*α*, IL-1, IL-6, and IL-8 (52–55).

Here, we show that flavonols, specifically quercetin, fisetin, and myricetin, are linked to a significant reduction in JCPyV infection as observed in both SV40 transformed glial cells (SVG-As) and in normal human astrocytes (NHAs). We next observed the time frame in which flavonols would be able to inhibit JCPyV infection. Finally, we showed that flavonols could inhibit JCPyV spread in an already established infection, suggesting that they may be a viable therapeutic for the treatment of PML.

## MATERIALS AND METHODS

### Cells and virus

 SVG-A cells were grown in 1× minimum essential media ([MEM], Corning) supplemented with 10% fetal bovine serum (Atlanta Biologics) and 1% Antibiotic-Antimycotic (Thermo Fisher). Primary NHAs were grown in NHA Media (AM; ScienceCell), supplemented with 2% fetal bovine serum (ScienCell), 1% astrocyte growth supplement (ScienCell), and 1% penicillin/streptomycin (ScienCell). All cells were stored in a humidified incubator at 37°C with 5% CO_2_. Purified JCPyV was derived from the MAD1/SVEΔ strain and used for all infections in both SVG-A and NHA cells. The methodology for viral propagation and purification has been described (56).

### Compounds

Quercetin was purchased from Tocris. Fisetin and myricetin were purchased from MedChem Express. All compounds were diluted in DMSO (Sigma Aldrich) and aliquots were stored at −80°C in single-use aliquots, according to the manufacturing protocol until use.

### Toxicity assays

SVG-A cells were plated at 5,000 cells per well, and NHAs were plated at 10,000 per well in a Corning 96-well tissue culture-treated plate. The following day, the cells were treated with a twofold dilution series of either quercetin, myricetin, or fisetin, ranging from 20 to 0.15625 µM, in phenol-free media. SVG-A cells were incubated with drug for 72 h in the incubator at 37°C with 5% CO_2_. NHA cells were incubated with drug for 120 h at 37°C with 5% CO_2_ to match the exposure times for infection. After incubation, a luciferase-based Viral ToxGlo Assay (Promega) was added to each well according to the manufacturer’s protocol. Viral ToxGlo measures the amount of ATP activity present through a luminescent signal. The cells were incubated with Viral ToxGlo lytic reagent for 10 min at 37°C with 5% CO_2_. Lysed cell suspension was then transferred to an opaque plate, and luciferase activity was read on the Glomax Multi-detection System (Promega, 0.5 s integration time) and normalized to a vehicle control.

### Infection assays

SVG-A cells were plated at 5,000 cells per well, and NHAs were plated at 10,000 per well. The following day, the cells were treated with the above-described dose curve of either quercetin, fisetin, or myricetin, ranging from 10 to 0.15625 µM. A DMSO (vehicle) control that was volume matched to the highest volume of each flavonol was included. The plates were pretreated with drug for 2 h at 37°C with 5% CO_2_. Next, the cells were incubated in 1× MEM with purified JCPyV (MOI of 10) in the continued presence of either quercetin, myricetin, fisetin, or a DMSO vehicle control matched to the highest concentration of each flavonol. Plates were incubated for 2 h at 37°C with 5% CO_2_ and rocked every 15 min to ensure homogenous distribution of virus in each well. Virus-containing media were then aspirated and replaced with either quercetin, myricetin, fisetin, or a volume-matched DMSO control in complete media. SVG-A cells were incubated for 72 h in the incubator at 37°C with 5% CO_2_. Astrocytes were incubated for 120 h at 37°C with 5% CO_2_. Plates were fixed in 100% ice-cold methanol and quantified using indirect immunofluorescence staining for the late viral protein VP1.

### Infection time course

SVG-A cells were plated at 5,000 cells per well, and NHAs were plated at 10,000 per well in a Corning 96-well tissue culture-treated plate. The following day, the cells were treated at the following four time points: 2 h before infection (−2 h; pretreatment), 0 h (co-treatment at the time of infection), 2 hs after infection (post treatment), or 24 h after infection (post-treatment). At the 2-h pretreatment time point, quercetin, myricetin, fisetin, or a volume-matched DMSO control was added in complete media. For all other time points, complete media were administered as a mock treatment. The plates were incubated for 2 h at 37°C and 5% CO_2_. At the 0-h time point, purified JCPyV (MOI of 10) in unsupplemented media was added to each of the time points, either alone or in the presence of drug, as appropriate for each treatment condition. In the −2 h and 0 h treated wells, quercetin, myricetin, fisetin, or a volume-matched DMSO control was added as co-treatment. All plates were infected with purified JCPyV at the same time, for 2 h at 37°C and 5% CO_2_. At the 2-h time point, the virus was aspirated and flavonols in complete media were added to the −2, 0, and 2 h time points. Complete media without drug or vehicle control was administered to the 24-h time point. Twenty-four hours after infection, flavonols were added to the 24-h time point, in complete media. SVG-A cells were incubated at 37°C and 5% CO_2_ for 72 h after infection, and NHA cells were incubated at 37°C and 5% CO_2_ for 120 h after infection. Plates were then stained and infection was quantified via indirect immunofluorescence.

### Viral spread assay

For the viral spread assay, the cells were plated at a density of 5,000 cells per well for SVG-A in complete MEM, or 10,000 cells per well for NHA in complete AM, and incubated overnight at 37°C with 5% CO_2_. The next day, the cells were infected with purified JCPyV (MOI of 10) for 2 h to establish an infection without the presence of drugs. After infection, JCPyV media were aspirated and plates were incubated with complete media as required for each cell type. One day post-infection, the cells were incubated with the highest nontoxic dose (for SVG-A: 10 µM quercetin, 10 µM myricetin, 10 µM fisetin; for NHA: 5 µM quercetin, 10 µM myricetin, 5 µM fisetin) or a volume-matched DMSO control. Plates were incubated at 37°C with 5% CO_2_ and infection was measured by indirect immunofluorescence quantification at 3, 6, and 9 days post-infection in SVG-As or 5, 10, and 15 days post-infection for NHAs. At every time point, the cells were either fixed in ice-cold methanol or were supplemented with an additional 50 µL of cell media that consisted of the matched concentration of quercetin, fisetin, myricetin, or DMSO vehicle control. Cell counts were obtained by counting the total number of cells in 10 random fields per well (3 wells total per replicate) on the Ti2-E Inverted Fluorescent Microscope (Nikon).

### Indirect immunofluorescence

To quantify the number of infected cells, SVG-A and NHA cells were fixed with ice-cold 100% methanol at −20°C for at least 15 min to permeabilize cells. The cells were rehydrated with 1× phosphate-buffered saline (PBS) for 15 min and rocked at room temperature. Subsequent permeabilization was conducted with incubation in 1% Triton-X (USB) for 10 min with rocking at room temperature. After washing with 1× PBS, cells underwent a 1-h incubation in PAb597, a primary antibody for VP1, diluted at 1:50 in 1× PBS. The cells were washed in 1× PBS and then incubated for 1 h in the dark in Goat Anti-mouse Alexa Fluor 488 conjugated antibody (Thermo Fisher Scientific) for 1 h, using a 1:500 dilution in 1× PBS. The cells were washed in PBS and then counterstained with 4′-6 diamidino-2-phenylindole (DAPI) diluted 1:1,000 in 1× PBS for 5 min in the dark. Following incubation, DAPI was removed, and the cells were washed extensively in PBS to remove residual stain. The cells were imaged on a Ti2-E Inverted Fluorescence Microscope (Nikon) using the 20× objective. For each plate, a random subset of 10 fields was chosen to image per well, and three wells were analyzed per condition. Images were analyzed using Elements High Content Imaging software (Nikon) to count total cells versus VP1+ cells.

### Statistics

A linear regression between successive points was used to calculate the half maximal inhibitory values (IC50) from the dose curves of each drug. Infections were normalized to a DMSO vehicle control. Significance was calculated using Student’s two-tailed *t*-test in GraphPad Prism. Values of *P* < 0.05 were considered significant. Data represent three independent experiments, each in triplicate, unless otherwise noted.

## RESULTS

### Quercetin, myricetin, and fisetin inhibit JCPyV infection in SVG-A cells

**Fig 2:**
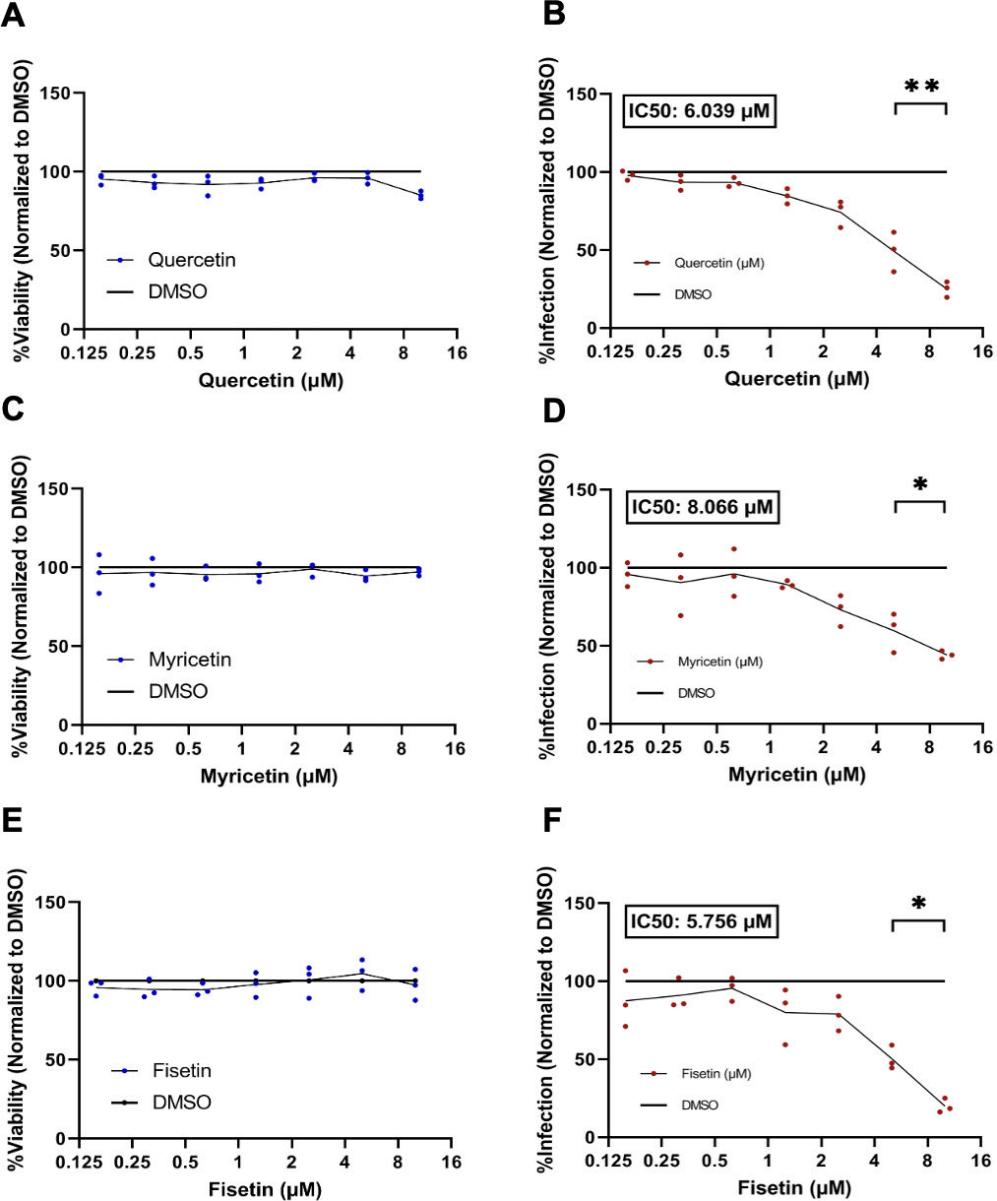
The cytotoxicity of quercetin (**A**), myricetin (**C**), and fisetin (**E**) was monitored after 72 h of incubation in SVG-A cells and quantified by a luciferase assay, and the results were normalized to a DMSO vehicle control that was volume matched to the highest volume of drug. SVG-A cells were incubated with quercetin (**B**), myricetin (**D**), or fisetin (**F**) for 2 h prior to JCPyV infection, and the cells were maintained in the presence of flavonol for 72 h. Infection of SVG-A cells was quantified by indirect immunofluorescence for JCPyV VP1+ signal. IC50 values are provided for quercetin, myricetin, and fisetin. Toxicity assays were run via three replicates in triplicate; data points in panels A, C, and E represent the mean of three wells each. Infection experiments were conducted with three replicates in triplicate; data points in panels B, D, and F represent the mean of each replicate. *P < 0.05 and **P < 0.01 calculated by Student’s two-tailed *t*-test.

Cytotoxicity of quercetin, myricetin, and fisetin was determined over a range of doses using a luciferase-based ViralToxGlo Assay (Promega). Toxicity values were obtained via three replicates in triplicate. Data points in panels A, C, and E represent three wells each. Quercetin, myricetin, and fisetin were both well tolerated by SVG-A cells with no significant change in viability compared to the vehicle-treated control at concentrations up to 10 µM (Fig. 2A, C, and E). All three drugs up to 10 µM were used to assess their effect on initial viral infection by JCPyV by measuring VP1 expression at 72 h post-infection. Quercetin, myricetin, and fisetin significantly decreased JCPyV infection (Fig. 2B, D, and F). A linear line of best fit was used to calculate the half-maximal inhibitory value (IC50) from the dose-dependent curves for each of the flavonols. Infection experiments were conducted with three replicates in triplicate. Data points in panels B, D, and E represent the means of three independent experiments. *P < 0.05 and **P < 0.01 calculated by Student’s two-tailed *t*-test.

### Quercetin, myricetin, and fisetin inhibit JCPyV infection in NHA cells

**Fig 3:**
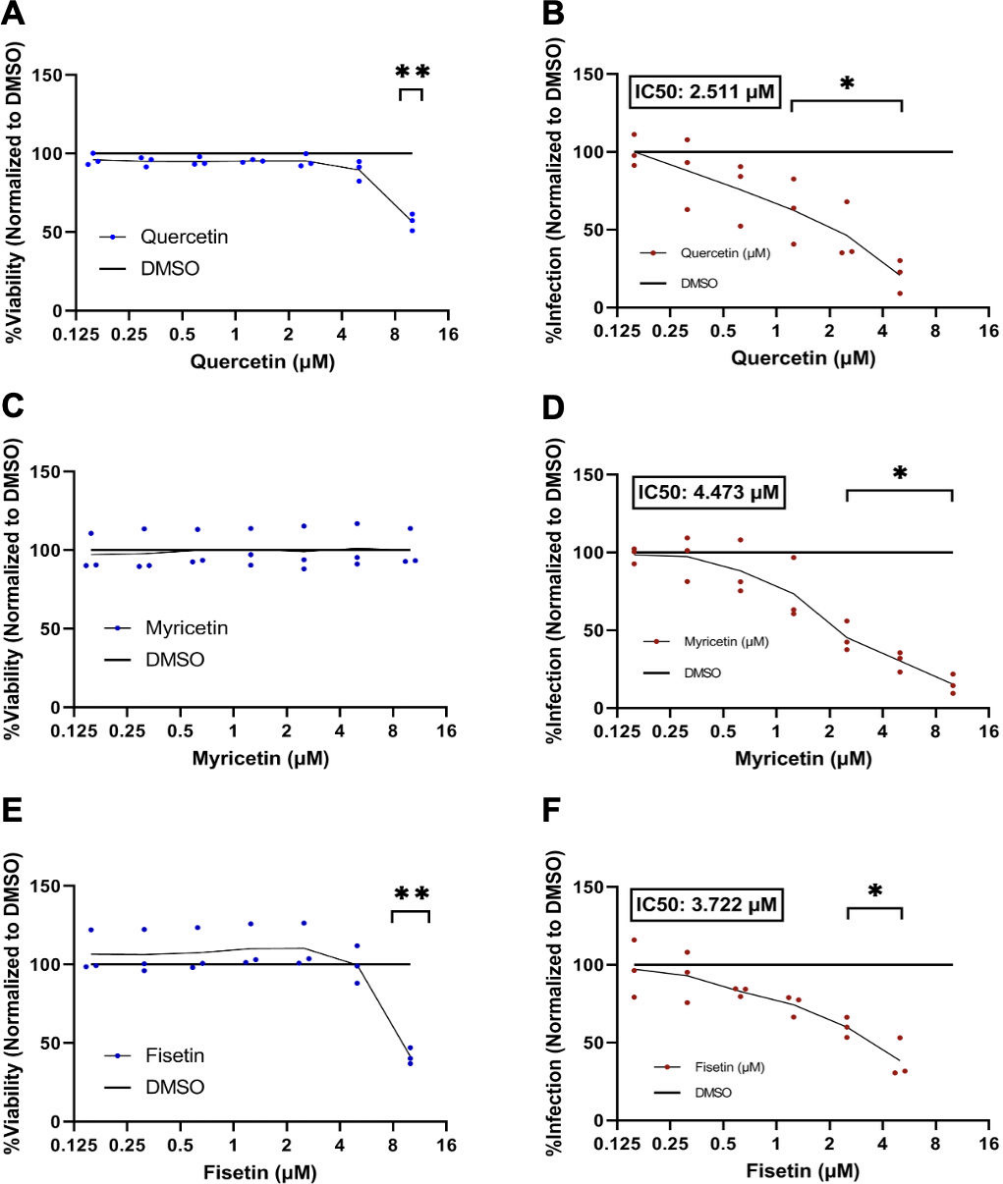
The cytotoxicity of quercetin (**A**), myricetin (**C**), or fisetin (**E**) was monitored after 120 h of incubation in NHA cells and quantified by a luciferase assay. Results were normalized to a DMSO vehicle control that was volume matched to the highest volume of drug. (**D**) NHA cells were incubated with quercetin (**B**), myricetin (**D**), or fisetin (**F**) 2 h prior to JCPyV infection, and the cells were maintained in media with drug for 120 h. Infected NHA cells were quantified by indirect immunofluorescence. IC50 values are provided for quercetin, myricetin, and fisetin. Toxicity assays were run via three replicates in triplicate; data points in panels A, C, and E represent the mean of three wells each. Infection experiments were conducted with three replicates in triplicate; data points in panels B, D, and F represent the mean of each replicate. *P < 0.05, **P < 0.01, and ***P < 0.001 calculated by Student’s two-tailed *t*-test.

In addition to SVG-A cells, we examined the effect of flavonols on JCPyV infection of primary NHAs. Myricetin was well tolerated in NHA with no significant change in viability up to a concentration of 10 µM (Fig. 3C) but both quercetin and fisetin were more toxic in NHA compared to SVG-A (Fig. 3A and E). Toxicity values were obtained via three replicates in triplicate. Data points in panels A, C, and E represent one well each. NHA were then treated with each drug up to 5 µM (quercetin and fisetin) or 10 µM (myricetin) for 2 h and then challenged with JCPyV. Infection was scored by assessing VP1 expression at 120 h post-infection. All flavonols were able to reduce JCPyV infection in a dose-dependent manner (Fig. 2F and 3B and D). IC50 values were calculated via a linear line of best fit. IC50 values were lower in NHAs than for SVG-As for each flavonol. Infection experiments were conducted with three replicates in triplicate. Data points in panels B, D, and E represent the means of three independent experiments. *P < 0.05, **P < 0.01, and ***P < 0.001 calculated by Student’s two-tailed *t*-test.

### Early administration of quercetin and fisetin suppresses JCPyV replication in SVG-A and NHA cells

**Fig 4:**
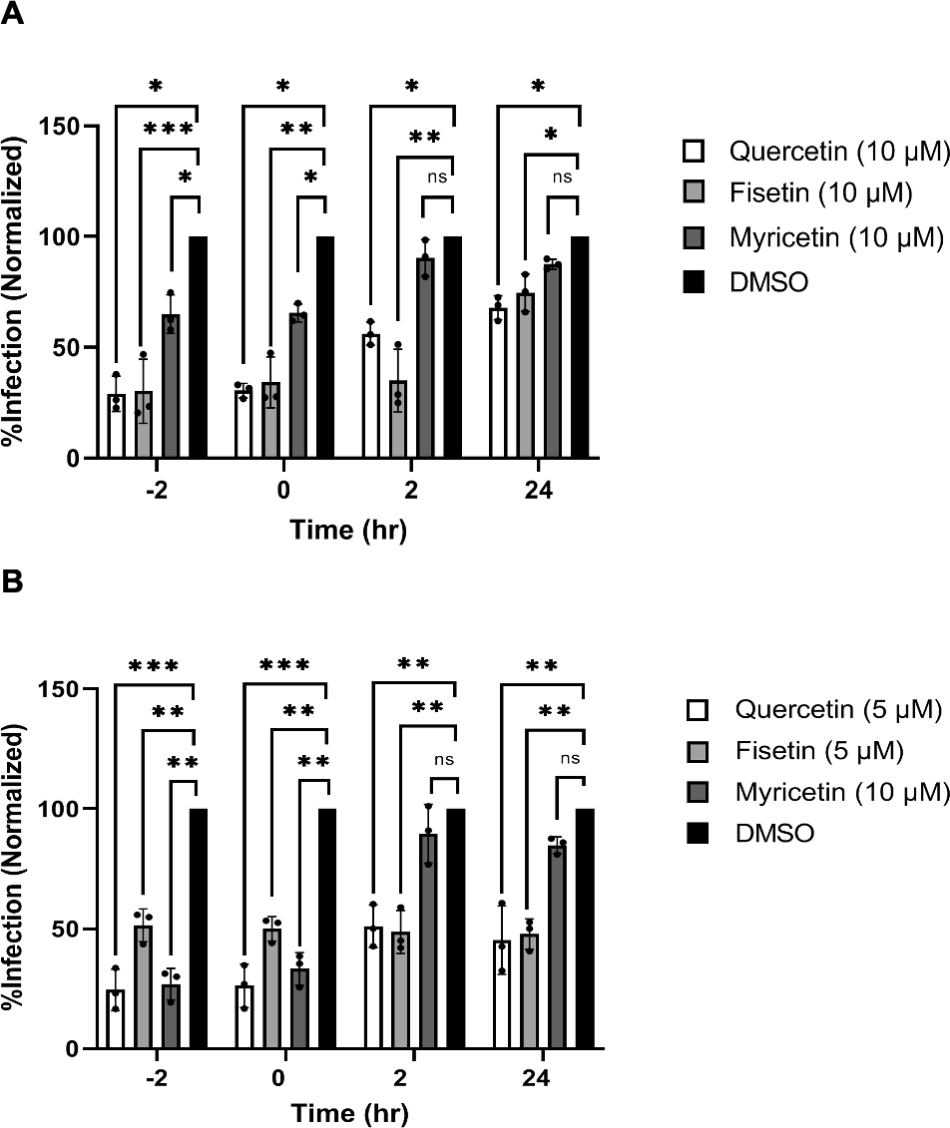
(**A**) SVG-A cells were administered 10 µM quercetin, myricetin, or fisetin at various time points in relation to JCPyV infection. After 72 hpost-infection, cultures were quantified for JCPyV VP1+ expression. (**B**) NHA cells were administered 5 µM quercetin, 10 µM myricetin, or 5 µM fisetin at various time points in relation to JCPyV infection. At 120 h post-infection, cultures were quantified for JCPyV VP1+ expression. Data points were normalized to a volume-matched DMSO control and represent three replicates of each experiment conducted in triplicate. *P < 0.05, **P < 0.01, and ***P < 0.001 calculated by Student’s two-tailed *t*-test. NS = not significant. Overall significance was determined through testing the significance of each individual replicate to the DMSO vehicle control. Error bars represent the standard deviation.

To determine what stage in the viral life cycle was being inhibited by flavonols, we treated cells with drug at multiple time points pre- and post-infection. Infectivity was measured by indirect immunofluorescence of JCPyV VP1. In SVG-A cells, quercetin and fisetin inhibited infection at all time points up to and including 24 h post-infection (Fig. 4A). The effect of myricetin on SVG-A cells was less pronounced and effective only when given 2 h before infection or at the time of infection (Fig. 4A). In NHAs, quercetin and fisetin maintained their ability to inhibit infection even when administered at 24 h post-infection (Fig. 4B). The effect of myricetin was again lost by 2 h post-infection (Fig. 4B).

### Quercetin and fisetin suppress viral spread during an ongoing JCPyV infection

**Fig 5:**
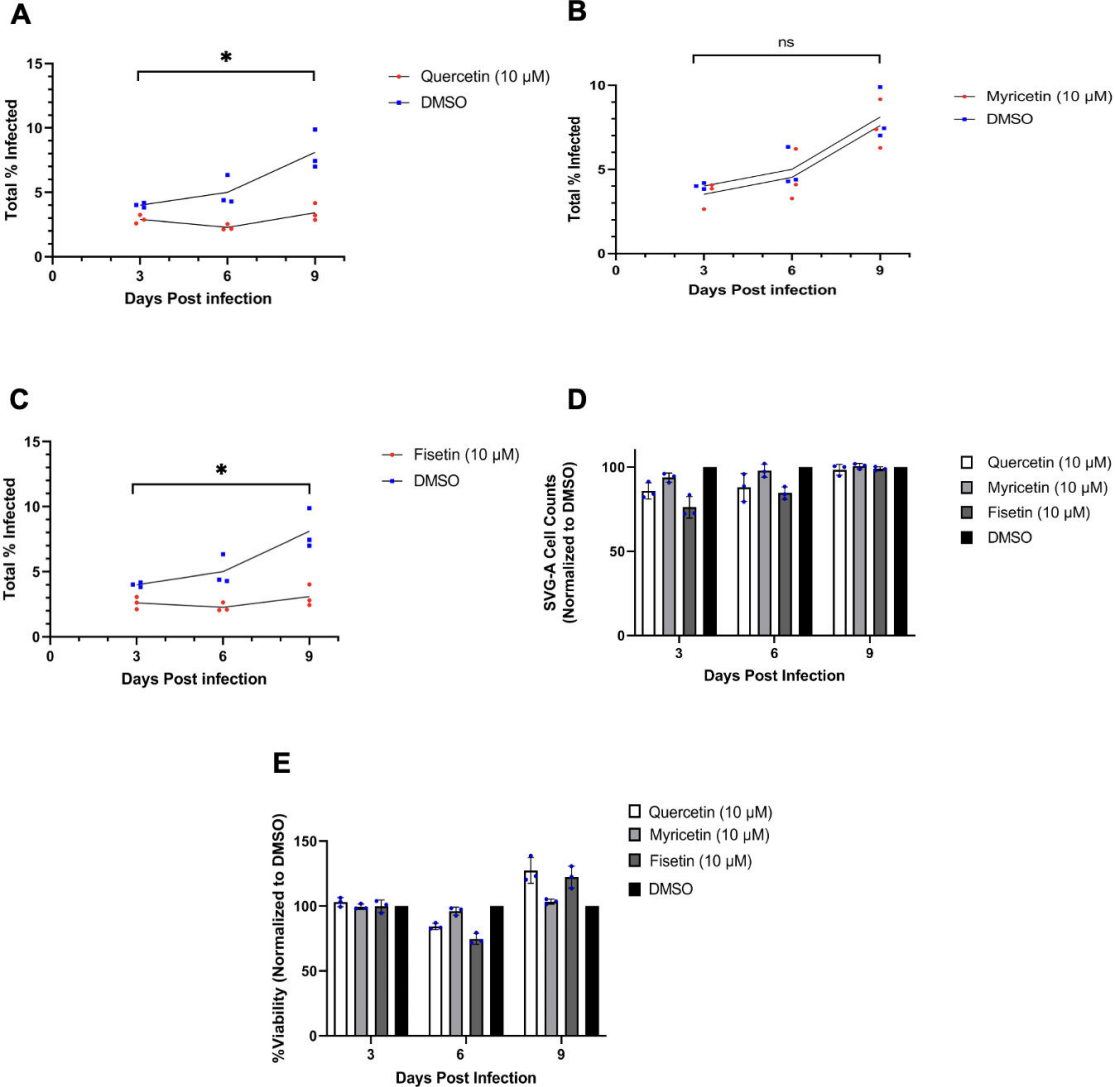
SVG-A cells were incubated with virus and the infection was allowed to establish for 24 h before flavonol treatment. Quercetin (**A**), myricetin (**B**), and fisetin (**C**) were administered at days 3, 6, and 9. Results were quantified at days 3, 6, and 9 by indirect immunofluorescence for VP1+ expression. Cell counts (**D**) and cytotoxicity (**E**) were also determined at each respective time point post-infection. Data points represent three replicates of each experiment conducted in triplicate. *P < 0.05, ns = not significant calculated by Student’s two-tailed *t*-test.

**Fig 6:**
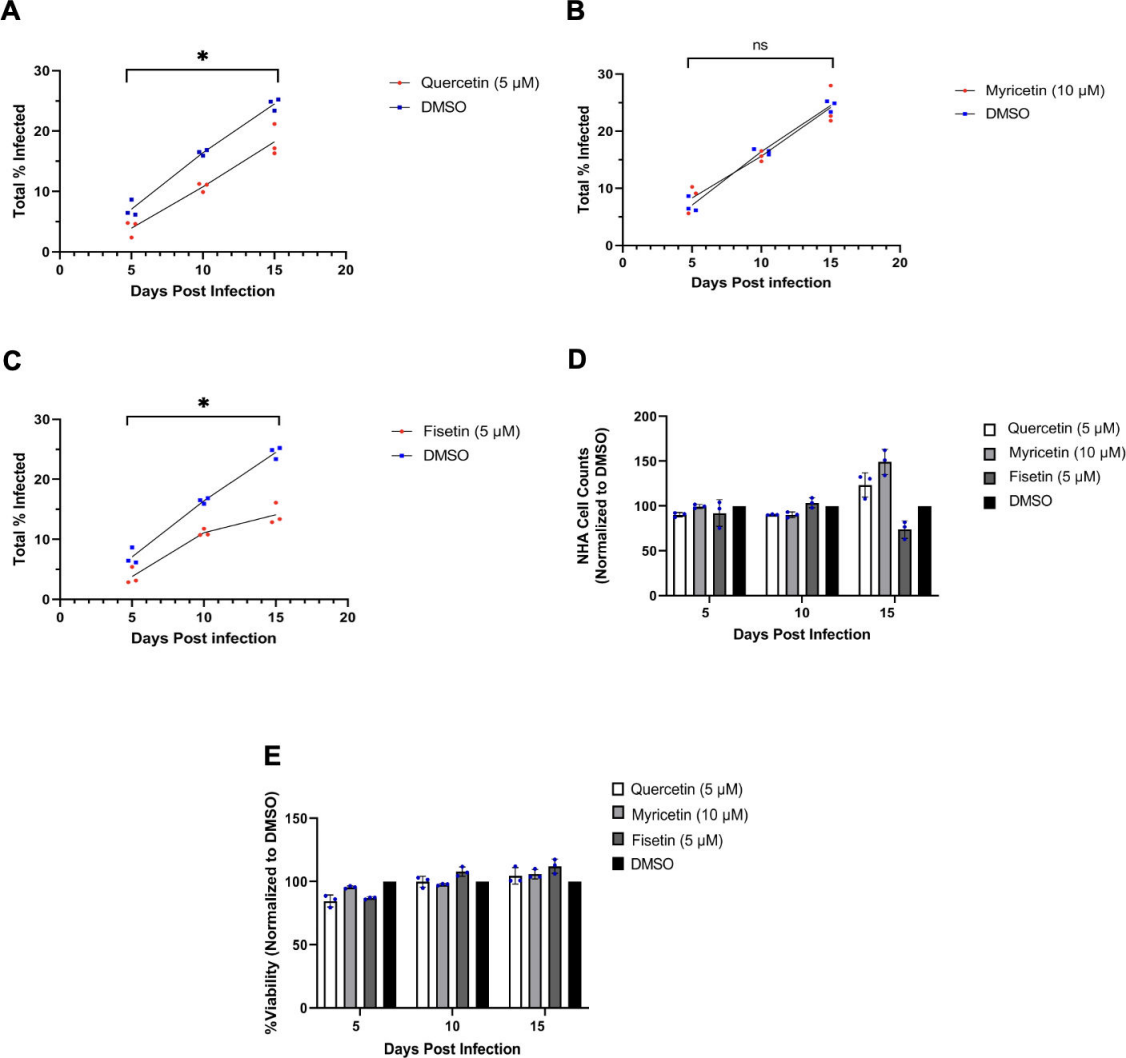
NHA cells were incubated with JCPyV, and the infection was allowed to establish for 24 h before flavonol treatment. Supplemental quercetin (**A**), myricetin (**B**), and fisetin (**C**) were administered at days 5, 10, and 15. Results were quantified on days 5, 10, and 15 by indirect immunofluorescence for VP1+ expression. Cell counts (**D**) and cytotoxicity (**E**) were also determined at each respective time point post-infection. *P < 0.05, ns = not significant calculated by Student’s two-tailed *t*-test. Data points represent the means of three independent experiments, each in triplicate.

We next asked whether these drugs could suppress an already established infection. SVG-A and NHA cells were first infected with JCPyV, and the infection was allowed to proceed for 24 h before administration of flavonols. Quercetin and fisetin were found to inhibit viral spread in SVG-A cells at 3, 6, and 9 days post-infection (Fig. 5A and C). As expected, myricetin showed no significant reduction in viral spread compared to DMSO control (Fig. 5B). In NHA, quercetin and fisetin were found to significantly reduce JCPyV infection at all time points (Fig. 6A and C). Myricetin was without effect (Fig. 6B). Experimental controls for cytotoxicity and cytostatic effects were measured by determining cell counts over time and by the ViralToxGlo Assay (Fig. 5 and 6D and E, respectively).

## DISCUSSION

In this study, we show that the flavonols quercetin, myricetin, and fisetin suppressed JCPyV infection in SVG-A and NHA cells. We first demonstrated that each flavonol was able to significantly suppress JCPyV infection when SVG-A and NHA cells were treated prior to infection. Next, we identified the time points at which flavonol administration could suppress JCPyV infection. Specifically, quercetin and fisetin were able to significantly inhibit viral infection in SVG-A cells when cells were treated 2 h before infection and up to 24 h after infection. Similarly, quercetin and fisetin inhibited infection at all four time points (−2 h to +24 h) in NHA cells. Myricetin was found to suppress initial infection by JCPyV but was unable to significantly inhibit viral spread in an already established infection in both SVG-A and NHA cells. In contrast, both fisetin and quercetin were capable of inhibiting viral spread in an already ongoing infection. This difference may suggest that the additional hydroxyl groups around the A/B phenol rings of the flavonol core structure of myricetin may impact protein binding affinities at inhibitory targets post-JCPyV infection.

Many groups have highlighted the anti-inflammatory and anti-proliferative effects of flavonols as their mechanism of action for therapeutic potential (57–60). JCPyV infiltration in the brain has been shown to cause a lytic infection in astrocytes that leads to necrosis (61). Cell necrosis is frequently linked to pro-inflammatory cytokine release of IL-1, which is suggested to be involved in necrosis-induced inflammation (62). In the brain, high mobility group box 1 protein (HMGG1) released from necrotic neurons during neurodegeneration leads to neuroinflammation caused by M1 microglia. M1 microglia secrete pro-inflammatory cytokines including TNF-α, IL-1β, and IFN-γ*,* which drive secondary and tertiary neuronal necrosis (63). Studies have shown that pro-inflammatory cytokine expression of TNF-α induces both early and late JCPyV transcription due to JCPyV regulation by the kB control element (64). This information suggests that necrosis and associated neural inflammation are the drivers for neurodegeneration and JCPyV transcription. Flavonols have been shown to have apoptotic effects in cancer tumors through inhibition of the PI3K/AKT/mTOR pathway and stimulation of apoptosis in cancer cells as described. As to why infection results were more potent with flavonol administration to NHAs than SVG-As, it has been found in a previous study that the PI3K/AKT/mTOR pathway is required for JCPyV infection in NHAs compared to SVG-A cells due to NHAs lacking large T antigen (65). Thus, inhibiting this pathway is predicted to decrease infection in the NHA cell line. Quercetin, myricetin, and fisetin have been found to competitively bind to the ATP pocket of PI3K and inhibit function (66–68). Myricetin has also been found to directly bind to the ATP binding pocket of AKT, and fisetin has been found to directly bind to the ATP binding pocket of mTOR (68, 69). Stimulating apoptosis due to direct inhibition of survival pathways may prevent inflammation stemming from a necrotic state within the brain leading to neuroprotection and decreased morbidity (70). Extensive studies have additionally shown that flavonols have inhibitory effects on the NF-kB pathway and indirectly suppress TNF-α with an associated decrease in JCPyV transcription effects (71).

Additionally, JCPyV infection has a link to ROS production in cells with an alternative pathway of initiating transcription. Specifically, JCPyV infection has been shown to lead to increased HIF-α translocation to the nucleus, which occurs due to an increase in oxidative stress. This increase in oxidative stress has been linked to increased JCPyV transcription levels (72). The tested flavonols have been identified in past studies to inhibit the production of free oxygen radicals and oxidative stress within the cells and serve as potent therapeutic antioxidants with extensive research in many chronic diseases (73–76).

The remaining question that persists is whether flavonols can effectively cross the BBB to work effectively as a therapeutic. Flavonoids have been shown to cross the BBB in various *in vitro* and *in vivo* models (77, 78). Specifically, fisetin was found to cross the BBB *in vivo* causing amplification of hippocampal long-term potentiation in neurons displaying significant penetration into the brain (79). Quercetin has been shown to cross the BBB in *in vitro* models with up to 65% crossing in U251 cells in addition to rat models (78, 80). Further, many groups have experimented with nanoparticle extracellular vesicle testing or quercetin co-administration with alpha-tocopherol with positive effects in crossing the BBB *in vitro* (81, 82). There are no published data on whether myricetin can cross the BBB, but some studies have shown the potential for myricetin to decrease endothelial cell permeability and inflammation as conducted in tissue culture models of a BBB (83).

One limitation in this study is that although the mechanism of flavonols has been identified in other models and cell types, the mechanism of action of these flavonols is unknown in the context of JCPyV and remains a subject of future research. Additionally, models describing flavonols crossing the BBB (except in the case of quercetin) have involved mainly *in vitro* or animal studies and have not been definitively proven to cross the BBB in humans (78–82). Thus, there is no definitive proof that flavonols will cross the BBB in patients. However, flavonols offer a novel route for suppressing JCPyV infection and spread, and they are exciting new drugs to be further investigated as a therapeutic for treating patients suffering from or at risk for developing PML.

## Data Availability

All data will be made available without restriction upon request.

## References

[1] Assetta B, Atwood WJ. 2017. The biology of JC polyomavirus. Biol Chem 398:839–855. doi:10.1515/hsz-2016-0345 28493815

[2] Tan CS, Koralnik IJ. 2010. Progressive multifocal leukoencephalopathy and other disorders caused by JC virus: clinical features and pathogenesis. Lancet Neurol 9:425–437. doi:10.1016/S1474-4422(10)70040-5 20298966 PMC2880524

[3] Atkinson AL, Atwood WJ. 2020. Fifty years of JC polyomavirus: a brief overview and remaining questions. Viruses 12:969. doi:10.3390/v12090969 32882975 PMC7552028

[4] Cortese I, Reich DS, Nath A. 2021. Progressive multifocal leukoencephalopathy and the spectrum of JC virus-related disease. Nat Rev Neurol 17:37–51. doi:10.1038/s41582-020-00427-y 33219338 PMC7678594

[5] Bofill-Mas S, Girones R. 2001. Excretion and transmission of JCV in human populations. J Neurovirol 7:345–349. doi:10.1080/13550280152537210 11517414

[6] Bofill-Mas S, Girones R. 2003. Role of the environment in the transmission of JC virus. J Neurovirol 9 Suppl 1:54–58. doi:10.1080/13550280390195306 12709873

[7] Kitamura T, Sugimoto C, Kato A, Ebihara H, Suzuki M, Taguchi F, Kawabe K, Yogo Y. 1997. Persistent JC virus (JCV) infection is demonstrated by continuous shedding of the same JCV strains. J Clin Microbiol 35:1255–1257. doi:10.1128/jcm.35.5.1255-1257.1997 9114418 PMC232740

[8] Bayliss J, Karasoulos T, McLean CA. 2013. Immunosuppression increases JC polyomavirus large T antigen DNA load in the brains of patients without progressive multifocal leukoencephalopathy. J Infect Dis 207:133–136. doi:10.1093/infdis/jis668 23112281

[9] Rindi LV, Zaçe D, Braccialarghe N, Massa B, Barchi V, Iannazzo R, Fato I, De Maria F, Kontogiannis D, Malagnino V, Sarmati L, Iannetta M. 2024. Drug-induced progressive multifocal leukoencephalopathy (PML): a systematic review and meta-analysis. Drug Saf 47:333–354. doi:10.1007/s40264-023-01383-4 38321317

[10] Ye D, Zimmermann T, Demina V, Sotnikov S, Ried CL, Rahn H, Stapf M, Untucht C, Rohe M, Terstappen GC, Wicke K, Mezler M, Manninga H, Meyer AH. 2021. Trafficking of JC virus-like particles across the blood-brain barrier. Nanoscale Adv 3:2488–2500. doi:10.1039/d0na00879f 36134165 PMC9418390

[11] O’Hara BA, Lukacher AS, Garabian K, Kaiserman J, MacLure E, Ishikawa H, Schroten H, Haley SA, Atwood WJ. 2024. Highly restrictive and directional penetration of the blood cerebral spinal fluid barrier by JCPyV. PLoS Pathog 20:e1012335. doi:10.1371/journal.ppat.1012335 39038049 PMC11293668

[12] Kondo Y, Windrem MS, Zou L, Chandler-Militello D, Schanz SJ, Auvergne RM, Betstadt SJ, Harrington AR, Johnson M, Kazarov A, Gorelik L, Goldman SA. 2014. Human glial chimeric mice reveal astrocytic dependence of JC virus infection. J Clin Invest 124:5323–5336. doi:10.1172/JCI76629 25401469 PMC4348956

[13] Zurhein G, Chou SM. 1965. Particles resembling Papova viruses in human cerebral demyelinating disease. Science 148:1477–1479. doi:10.1126/science.148.3676.1477 14301897

[14] Kim J, Kim C, Lee JA, Lee SJ, Lee KH, Kim JH, Ahn JY, Jeong SJ, Ku NS, Choi JY, Yeom JS, Song YG. 2023. Long-term prognosis and overall mortality in patients with progressive multifocal leukoencephalopathy. Sci Rep 13:14291. doi:10.1038/s41598-023-41147-9 37652945 PMC10471597

[15] Kim Jinnam, Kim CH, Lee JA, Lee SJ, Lee KH, Kim JH, Ahn JY, Jeong SJ, Ku NS, Choi JY, Yeom J, Song YG. 2022. 2311. Long-term prognosis and risk factors for overall mortality in patients with progressive multifocal leukoencephalopathy. Open Forum Infect Dis 9. doi:10.1093/ofid/ofac492.143 PMC10471597 37652945

[16] D’Souza A, Wilson J, Mukherjee S, Jaiyesimi I. 2010. Progressive multifocal leukoencephalopathy in chronic lymphocytic leukemia: a report of three cases and review of the literature. Clin Lymphoma Myeloma Leuk 10:E1–9. doi:10.3816/CLML.2010.n.009 20223720

[17] Astrom KE, Mancall EL, Richardson EP Jr. 1958. Progressive multifocal leuko-encephalopathy; a hitherto unrecognized complication of chronic lymphatic leukaemia and Hodgkin’s disease. Brain (Bacau) 81:93–111. doi:10.1093/brain/81.1.93 13523006

[18] Berger JR, Kaszovitz B, Post MJ, Dickinson G. 1987. Progressive multifocal leukoencephalopathy associated with human immunodeficiency virus infection. a review of the literature with a report of sixteen cases. Ann Intern Med 107:78–87. doi:10.7326/0003-4819-107-1-78 3296901

[19] Sidhu N, McCutchan JA. 2010. Unmasking of PML by HAART: unusual clinical features and the role of IRIS. J Neuroimmunol 219:100–104. doi:10.1016/j.jneuroim.2009.11.013 19962769 PMC2825275

[20] Shelburne SA 3rd, Hamill RJ, Rodriguez-Barradas MC, Greenberg SB, Atmar RL, Musher DW, Gathe JC Jr, Visnegarwala F, Trautner BW. 2002. Immune reconstitution inflammatory syndrome: emergence of a unique syndrome during highly active antiretroviral therapy. Medicine (Baltimore) 81:213–227. doi:10.1097/00005792-200205000-00005 11997718

[21] Post MJD, Thurnher MM, Clifford DB, Nath A, Gonzalez RG, Gupta RK, Post KK. 2013. CNS-immune reconstitution inflammatory syndrome in the setting of HIV infection, part 2: discussion of neuro-immune reconstitution inflammatory syndrome with and without other pathogens. AJNR Am J Neuroradiol 34:1308–1318. doi:10.3174/ajnr.A3184 22790252 PMC4905746

[22] Fournier A, Martin-Blondel G, Lechapt-Zalcman E, Dina J, Kazemi A, Verdon R, Mortier E, de La Blanchardière A. 2017. Immune reconstitution inflammatory syndrome unmasking or worsening AIDS-related progressive multifocal leukoencephalopathy: a literature review. Front Immunol 8:577. doi:10.3389/fimmu.2017.00577 28588577 PMC5440580

[23] Pavlovic D, Patera AC, Nyberg F, Gerber M, Liu M, Progressive Multifocal Leukeoncephalopathy C. 2015. Progressive multifocal leukoencephalopathy: current treatment options and future perspectives. Ther Adv Neurol Disord 8:255–273. doi:10.1177/1756285615602832 26600871 PMC4643867

[24] Jiang ZG, Cohen J, Marshall LJ, Major EO. 2010. Hexadecyloxypropyl-cidofovir (CMX001) suppresses JC virus replication in human fetal brain SVG cell cultures. Antimicrob Agents Chemother 54:4723–4732. doi:10.1128/AAC.00837-10 20823288 PMC2976159

[25] Kaiserman J, O’Hara BA, Haley SA, Atwood WJ. 2023. An elusive target: inhibitors of JC polyomavirus infection and their development as therapeutics for the treatment of progressive multifocal leukoencephalopathy. Int J Mol Sci 24:8580. doi:10.3390/ijms24108580 37239927 PMC10218015

[26] Hall CD, Dafni U, Simpson D, Clifford D, Wetherill PE, Cohen B, McArthur J, Hollander H, Yainnoutsos C, Major E, Millar L, Timpone J. 1998. Failure of cytarabine in progressive multifocal leukoencephalopathy associated with human immunodeficiency virus infection. AIDS Clinical Trials Group 243 Team. N Engl J Med 338:1345–1351. doi:10.1056/NEJM199805073381903 9571254

[27] Marra CM, Rajicic N, Barker DE, Cohen BA, Clifford D, Donovan Post MJ, Ruiz A, Bowen BC, Huang ML, Queen-Baker J, Andersen J, Kelly S, Shriver S, Adult ACTGT. 2002. A pilot study of cidofovir for progressive multifocal leukoencephalopathy in AIDS. AIDS 16:1791–1797. doi:10.1097/00002030-200209060-00012 12218391

[28] Kraemer C, Evers S, Nolting T, Arendt G, Husstedt IW. 2008. Cidofovir in combination with HAART and survival in AIDS-associated progressive multifocal leukoencephalopathy. J Neurol 255:526–531. doi:10.1007/s00415-008-0731-z 18202814

[29] Gosert R, Rinaldo CH, Wernli M, Major EO, Hirsch HH. 2011. CMX001 (1-O-hexadecyloxypropyl-cidofovir) inhibits polyomavirus JC replication in human brain progenitor-derived astrocytes. Antimicrob Agents Chemother 55:2129–2136. doi:10.1128/AAC.00046-11 21402853 PMC3088264

[30] Epker JL, van Biezen P, van Daele PLA, van Gelder T, Vossen A, van Saase JLCM. 2009. Progressive multifocal leukoencephalopathy, a review and an extended report of five patients with different immune compromised states. Eur J Intern Med 20:261–267. doi:10.1016/j.ejim.2008.07.032 19393493

[31] Breedveld FC, Dayer JM. 2000. Leflunomide: mode of action in the treatment of rheumatoid arthritis. Ann Rheum Dis 59:841–849. doi:10.1136/ard.59.11.841 11053058 PMC1753034

[32] O’Hara BA, Gee GV, Haley SA, Morris-Love J, Nyblade C, Nieves C, Hanson BA, Dang X, Turner TJ, Chavin JM, Lublin A, Koralnik IJ, Atwood WJ. 2021. Teriflunomide inhibits JCPyV infection and spread in glial cells and choroid plexus epithelial cells. Int J Mol Sci 22:9809. doi:10.3390/ijms22189809 34575975 PMC8468119

[33] Epperla N, Medina-Flores R, Mazza JJ, Yale SH. 2014. Mirtazapine and mefloquine therapy for non-AIDS-related progressive multifocal leukoencephalopathy. WMJ 113:242–245. 25745699

[34] Trentalange A, Calcagno A, Ghisetti V, Atzori C, Busolli P, Bonora S, Imperiale D. 2016. Clearance of cerebrospinal fluid JCV DNA with mirtazapine in a patient with progressive multifocal leukoencephalopathy and sarcoidosis. Antivir Ther 21:633–635. doi:10.3851/IMP3032 26857363

[35] Jamilloux Y, Kerever S, Ferry T, Broussolle C, Honnorat J, Sève P. 2016. Treatment of progressive multifocal leukoencephalopathy with mirtazapine. Clin Drug Investig 36:783–789. doi:10.1007/s40261-016-0433-8 27401779

[36] Wu Z, Graf FE, Hirsch HH. 2021. Antivirals against human polyomaviruses: leaving no stone unturned. Rev Med Virol 31:e2220. doi:10.1002/rmv.2220 33729628

[37] Nelson CDS, Carney DW, Derdowski A, Lipovsky A, Gee GV, O’Hara B, Williard P, DiMaio D, Sello JK, Atwood WJ. 2013. A retrograde trafficking inhibitor of ricin and Shiga-like toxins inhibits infection of cells by human and monkey polyomaviruses. mBio 4:e00729-13. doi:10.1128/mBio.00729-13 24222489 PMC3892778

[38] Kaiserman J, O’Hara BA, Garabian K, Lukacher A, Haley SA, Atwood WJ. 2023. The oxindole GW-5074 inhibits JC Polyomavirus infection and spread by antagonizing the MAPK-ERK signaling pathway. mBio 14:e0358322. doi:10.1128/mbio.03583-22 36786589 PMC10127638

[39] Boots AW, Wilms LC, Swennen ELR, Kleinjans JCS, Bast A, Haenen GRMM. 2008. In vitro and ex vivo anti-inflammatory activity of quercetin in healthy volunteers. Nutrition 24:703–710. doi:10.1016/j.nut.2008.03.023 18549926

[40] Spiegel M, Andruniów T, Sroka Z. 2020. Flavones’ and flavonols’ antiradical structure–activity relationship—a quantum chemical study. Antioxidants (Basel) 9:461. doi:10.3390/antiox9060461 32471289 PMC7346117

[41] Hollman PCH. 2004. Absorption, bioavailability, and metabolism of flavonoids. Pharm Biol 42:74–83. doi:10.3109/13880200490893492

[42] Chagas M, Behrens MD, Moragas-Tellis CJ, Penedo GXM, Silva AR, Gonçalves-de-Albuquerque CF. 2022. Flavonols and flavones as potential anti-inflammatory, antioxidant, and antibacterial compounds. Oxid Med Cell Longev 2022:9966750. doi:10.1155/2022/9966750 36111166 PMC9470311

[43] Yahfoufi N, Alsadi N, Jambi M, Matar C. 2018. The immunomodulatory and anti-inflammatory role of polyphenols. Nutrients 10:1618. doi:10.3390/nu10111618 30400131 PMC6266803

[44] Zughaibi TA, Suhail M, Tarique M, Tabrez S. 2021. Targeting PI3K/Akt/mTOR pathway by different flavonoids: a cancer chemopreventive approach. Int J Mol Sci 22:12455. doi:10.3390/ijms222212455 34830339 PMC8621356

[45] Cho SY, Park SJ, Kwon MJ, Jeong TS, Bok SH, Choi WY, Jeong WI, Ryu SY, Do SH, Lee CS, Song JC, Jeong KS. 2003. Quercetin suppresses proinflammatory cytokines production through MAP kinases and NF-κB pathway in lipopolysaccharide-stimulated macrophage. Mol Cell Biochem 243:153–160. doi:10.1023/a:1021624520740 12619901

[46] Gu L, Li Z, Zhang X, Chen M, Zhang X. 2023. Identification of MAP Kinase Kinase 3 as a protein target of myricetin in non-small cell lung cancer cells. Biomed Pharmacother 161:114460. doi:10.1016/j.biopha.2023.114460 36870282

[47] Chou RH, Hsieh SC, Yu YL, Huang MH, Huang YC, Hsieh YH. 2013. Fisetin inhibits migration and invasion of human cervical cancer cells by down-regulating urokinase plasminogen activator expression through suppressing the p38 MAPK-dependent NF-κB signaling pathway. PLoS One 8:e71983. doi:10.1371/journal.pone.0071983 23940799 PMC3733924

[48] Suh DK, Lee EJ, Kim HC, Kim JH. 2010. Induction of G(1)/S phase arrest and apoptosis by quercetin in human osteosarcoma cells. Arch Pharm Res 33:781–785. doi:10.1007/s12272-010-0519-4 20512478

[49] Yang W, Su J, Li M, Li T, Wang X, Zhao M, Hu X. 2021. Myricetin induces autophagy and cell cycle arrest of HCC by inhibiting MARCH1-regulated Stat3 and p38 MAPK signaling pathways. Front Pharmacol 12:709526. doi:10.3389/fphar.2021.709526 34733155 PMC8558373

[50] Tsai YH, Lin JJ, Ma YS, Peng SF, Huang AC, Huang YP, Fan MJ, Lien JC, Chung JG. 2019. Fisetin inhibits cell proliferation through the induction of G(0)/G(1) phase arrest and caspase-3-mediated apoptosis in mouse leukemia cells. Am J Chin Med 47:841–863. doi:10.1142/S0192415X19500447 31096772

[51] Zhang HW, Hu JJ, Fu RQ, Liu X, Zhang YH, Li J, Liu L, Li YN, Deng Q, Luo QS, Ouyang Q, Gao N. 2018. Flavonoids inhibit cell proliferation and induce apoptosis and autophagy through downregulation of PI3Kγ mediated PI3K/AKT/mTOR/p70S6K/ULK signaling pathway in human breast cancer cells. Sci Rep 8:11255. doi:10.1038/s41598-018-29308-7 30050147 PMC6062549

[52] Chekalina N, Burmak Y, Petrov Y, Borisova Z, Manusha Y, Kazakov Y, Kaidashev I. 2018. Quercetin reduces the transcriptional activity of NF-kB in stable coronary artery disease. Indian Heart J 70:593–597. doi:10.1016/j.ihj.2018.04.006 30392493 PMC6204471

[53] Chen M, Chen Z, Huang D, Sun C, Xie J, Chen T, Zhao X, Huang Y, Li D, Wu B, Wu D. 2020. Myricetin inhibits TNF-α-induced inflammation in A549 cells via the SIRT1/NF-κB pathway. Pulm Pharmacol Ther 65:102000. doi:10.1016/j.pupt.2021.102000 33601000

[54] Léotoing L, Wauquier F, Guicheux J, Miot-Noirault E, Wittrant Y, Coxam V. 2013. The polyphenol fisetin protects bone by repressing NF-κB and MKP-1-dependent signaling pathways in osteoclasts. PLoS One 8:e68388. doi:10.1371/journal.pone.0068388 23861901 PMC3701685

[55] Liu T, Zhang L, Joo D, Sun SC. 2017. NF-κB signaling in inflammation. Signal Transduct Target Ther 2:17023 doi:10.1038/sigtrans.2017.23 29158945 PMC5661633

[56] Shen PS, Enderlein D, Nelson CDS, Carter WS, Kawano M, Xing L, Swenson RD, Olson NH, Baker TS, Cheng RH, Atwood WJ, Johne R, Belnap DM. 2011. The structure of avian polyomavirus reveals variably sized capsids, non-conserved inter-capsomere interactions, and a possible location of the minor capsid protein VP4. Virology (Auckland) 411:142–152. doi:10.1016/j.virol.2010.12.005 PMC3057058 21239031

[57] Al-Khayri JM, Sahana GR, Nagella P, Joseph BV, Alessa FM, Al-Mssallem MQ. 2022. Flavonoids as potential anti-inflammatory molecules: a review. Molecules 27:2901. doi:10.3390/molecules27092901 35566252 PMC9100260

[58] Maleki SJ, Crespo JF, Cabanillas B. 2019. Anti-inflammatory effects of flavonoids. Food Chem 299:125124. doi:10.1016/j.foodchem.2019.125124 31288163

[59] Ackland ML, van de Waarsenburg S, Jones R. 2005. Synergistic antiproliferative action of the flavonols quercetin and kaempferol in cultured human cancer cell lines. In Vivo 19:69–76. 15796157

[60] Sinha R, Srivastava S, Joshi A, Joshi UJ, Govil G. 2014. In-vitro anti-proliferative and anti-oxidant activity of galangin, fisetin and quercetin: role of localization and intermolecular interaction in model membrane. Eur J Med Chem 79:102–109. doi:10.1016/j.ejmech.2014.04.002 24727463

[61] Seth P, Diaz F, Tao-Cheng JH, Major EO. 2004. JC virus induces nonapoptotic cell death of human central nervous system progenitor cell-derived astrocytes. J Virol 78:4884–4891. doi:10.1128/jvi.78.9.4884-4891.2004 15078969 PMC387680

[62] Martin SJ. 2016. Cell death and inflammation: the case for IL-1 family cytokines as the canonical DAMPs of the immune system. FEBS J 283:2599–2615. doi:10.1111/febs.13775 27273805

[63] Homma H, Tanaka H, Fujita K, Okazawa H. 2024. Necrosis links neurodegeneration and neuroinflammation in neurodegenerative disease. Int J Mol Sci 25:3636. doi:10.3390/ijms25073636 38612448 PMC11012149

[64] Wollebo HS, Safak M, Del Valle L, Khalili K, White MK. 2011. Role for tumor necrosis factor-α in JC virus reactivation and progressive multifocal leukoencephalopathy. J Neuroimmunol 233:46–53. doi:10.1016/j.jneuroim.2010.11.013 21185609 PMC3074035

[65] Wilczek MP, Armstrong FJ, Mayberry CL, King BL, Maginnis MS. 2021. PI3K/AKT/mTOR signaling pathway is required for JCPyV infection in primary astrocytes. Cells 10:3218. doi:10.3390/cells10113218 34831441 PMC8624856

[66] Boo HJ, Yoon D, Choi Y, Kim Y, Cha JS, Yoo J. 2025. Quercetin: molecular insights into its biological roles. Biomolecules 15:313. doi:10.3390/biom15030313 40149849 PMC11940409

[67] Walker EH, Pacold ME, Perisic O, Stephens L, Hawkins PT, Wymann MP, Williams RL. 2000. Structural determinants of phosphoinositide 3-kinase inhibition by wortmannin, LY294002, quercetin, myricetin, and staurosporine. Mol Cell 6:909–919. doi:10.1016/s1097-2765(05)00089-4 11090628

[68] Lim JY, Lee JY, Byun BJ, Kim SH. 2015. Fisetin targets phosphatidylinositol-3-kinase and induces apoptosis of human B lymphoma Raji cells. Toxicol Rep 2:984–989. doi:10.1016/j.toxrep.2015.07.004 28962438 PMC5598213

[69] Kumamoto T, Fujii M, Hou DX. 2009. Akt is a direct target for myricetin to inhibit cell transformation. Mol Cell Biochem 332:33–41. doi:10.1007/s11010-009-0171-9 19504174

[70] Heckmann BL, Tummers B, Green DR. 2019. Crashing the computer: apoptosis vs. necroptosis in neuroinflammation. Cell Death Differ 26:41–52. doi:10.1038/s41418-018-0195-3 30341422 PMC6294765

[71] Choy KW, Murugan D, Leong XF, Abas R, Alias A, Mustafa MR. 2019. Flavonoids as natural anti-inflammatory agents targeting nuclear factor-kappa B (NFκB) signaling in cardiovascular diseases: a mini review. Front Pharmacol 10:1295. doi:10.3389/fphar.2019.01295 31749703 PMC6842955

[72] Piña-Oviedo S, Khalili K, Del Valle L. 2009. Hypoxia inducible factor-1 alpha activation of the JCV promoter: role in the pathogenesis of progressive multifocal leukoencephalopathy. Acta Neuropathol 118:235–247. doi:10.1007/s00401-009-0533-0 19360424 PMC2856344

[73] Zahra M, Abrahamse H, George BP. 2024. Flavonoids: antioxidant powerhouses and their role in nanomedicine. Antioxidants (Basel) 13:922. doi:10.3390/antiox13080922 39199168 PMC11351814

[74] Xu D, Hu MJ, Wang YQ, Cui YL. 2019. Antioxidant activities of quercetin and its complexes for medicinal application. Molecules 24:1123. doi:10.3390/molecules24061123 30901869 PMC6470739

[75] Huang WC, Wu SJ, Yeh KW, Huang TH, Liou CJ. 2024. Protective effects of myricetin on airway inflammation and oxidative stress in ovalbumin-induced asthma mice. J Nutr Biochem 123:109485. doi:10.1016/j.jnutbio.2023.109485 37844766

[76] Hassan SSU, Samanta S, Dash R, Karpiński TM, Habibi E, Sadiq A, Ahmadi A, Bungau S. 2022. Corrigendum: the neuroprotective effects of fisetin, a natural flavonoid in neurodegenerative diseases: focus on the role of oxidative stress. Front Pharmacol 13:1095648. doi:10.3389/fphar.2022.1095648 36506577 PMC9727536

[77] Youdim KA, Dobbie MS, Kuhnle G, Proteggente AR, Abbott NJ, Rice-Evans C. 2003. Interaction between flavonoids and the blood-brain barrier: in vitro studies. J Neurochem 85:180–192. doi:10.1046/j.1471-4159.2003.01652.x 12641740

[78] Youdim KA, Qaiser MZ, Begley DJ, Rice-Evans CA, Abbott NJ. 2004. Flavonoid permeability across an in situ model of the blood-brain barrier. Free Radic Biol Med 36:592–604. doi:10.1016/j.freeradbiomed.2003.11.023 14980703

[79] He WB, Abe K, Akaishi T. 2018. Oral administration of fisetin promotes the induction of hippocampal long-term potentiation in vivo. J Pharmacol Sci 136:42–45. doi:10.1016/j.jphs.2017.12.008 29317180

[80] Liu Y, Tang ZG, Lin Y, Qu XG, Lv W, Wang GB, Li CL. 2017. Effects of quercetin on proliferation and migration of human glioblastoma U251 cells. Biomed Pharmacother 92:33–38. doi:10.1016/j.biopha.2017.05.044 28528183

[81] Ferri P, Angelino D, Gennari L, Benedetti S, Ambrogini P, Del Grande P, Ninfali P. 2015. Enhancement of flavonoid ability to cross the blood-brain barrier of rats by co-administration with α-tocopherol. Food Funct 6:394–400. doi:10.1039/c4fo00817k 25474041

[82] Pei J, Kumarasamy RV, Jayaraman S, Kanniappan GV, Long Q, Palanisamy CP. 2025. Quercetin-functionalized nanomaterials: Innovative therapeutic avenues for Alzheimer’s disease management. Ageing Res Rev 104:102665. doi:10.1016/j.arr.2025.102665 39824363

[83] Zhang S, Hu X, Guo S, Shi L, He Q, Zhang P, Yu S, Zhao R. 2019. Myricetin ameliorated ischemia/reperfusion-induced brain endothelial permeability by improvement of eNOS uncoupling and activation eNOS/NO. J Pharmacol Sci 140:62–72. doi:10.1016/j.jphs.2019.04.009 31130510

